# Do some people with a prolonged disorder of consciousness experience pain? A clinically focused narrative review and synthesis

**DOI:** 10.1177/02692155251333540

**Published:** 2025-04-13

**Authors:** Derick T Wade, Andrew Hanrahan

**Affiliations:** 1 Centre for Movement, Occupation and Rehabilitation Sciences (MOReS), 6395 Oxford Brookes University, Oxford, UK; 2 Royal Hospital for Neuro-disability, London, UK

**Keywords:** Prolonged Disorder of Consciousness, pain, awareness, PDOC

## Abstract

**Objective:**

To investigate the hypothesis that people with a prolonged disorder of consciousness experience nociceptive pain.

**Method:**

A non-systematic literature review into the nature and neurophysiological basis of consciousness and pain likely function when someone has severe thalamocortical dysfunction; the behavioural manifestations of pain in people who cannot communicate; and how they relate to the experience.

**Findings:**

Consciousness depends on thalamocortical integrity and is judged clinically by establishing the person's behaviour depends on extracting or using meaning. The experience of pain is also deduced from a person's behaviour, including increased purposeless motor movements, facial expressions, non-verbal vocal expressions and physiological (autonomic) changes such as tachycardia and tear production. Extensive brainstem and midbrain networks are activated by pain, including autonomic networks. Given their early evolution and location, they likely resist damage. The networks appear intrinsically resilient, functioning when damaged unless the damage is severe.

**Synthesis:**

Someone with a prolonged disorder of consciousness usually has intransitive consciousness (arousal) that is not dependent on cortical cognitive processes and may have retained occurrent consciousness of mental states when aroused. Nociceptive stimuli elicit automatic but purposeless behaviours typically associated with pain. These behaviours are likely to be responses to this unpleasant mental state of occurrent consciousness that is limited to the time they show pain behaviours, with no memory of it.

**Conclusion:**

The unconscious person with a prolonged disorder of consciousness exhibiting pain behaviours in response to nociceptive stimuli likely experiences pain without analysing its significance; they are unlikely to anticipate or remember it.

## Introduction

Can people who are unconscious or in the ‘vegetative state’ perceive pain? In 1991, Michael McQuillen answered, ‘*Although by definition the unconscious patient cannot tell you that he perceives pain, available data suggest that he may; therefore, you cannot know that he doesn't’.*^1^

The question is not trivial. In a recent case before the UK Court of Protection^2^ that went to appeal,^3^ the possibility of a person's pain influenced the best interest decision; similar arguments about pain and the burden of care have influenced other cases.^4,5^ Observers are distressed by pain behaviours. Family members and the clinical team frequently discuss possible suffering of pain, especially when considering withdrawal of clinically assisted nutrition and hydration.

Several recent reviews have explored how to determine when someone experiences pain, focusing on identifying pathways and neurophysiological indicators of pain^6–8^ but did not investigate the question posed.

This paper investigates the hypothesis that people with a prolonged disorder of consciousness who exhibit behaviours indicating a response to nociceptive stimuli may also have an aversive experience associated with these observed pain behaviours. We discuss the nature of the experience and give practical clinical conclusions based on relevant evidence.

**Figure 1.**
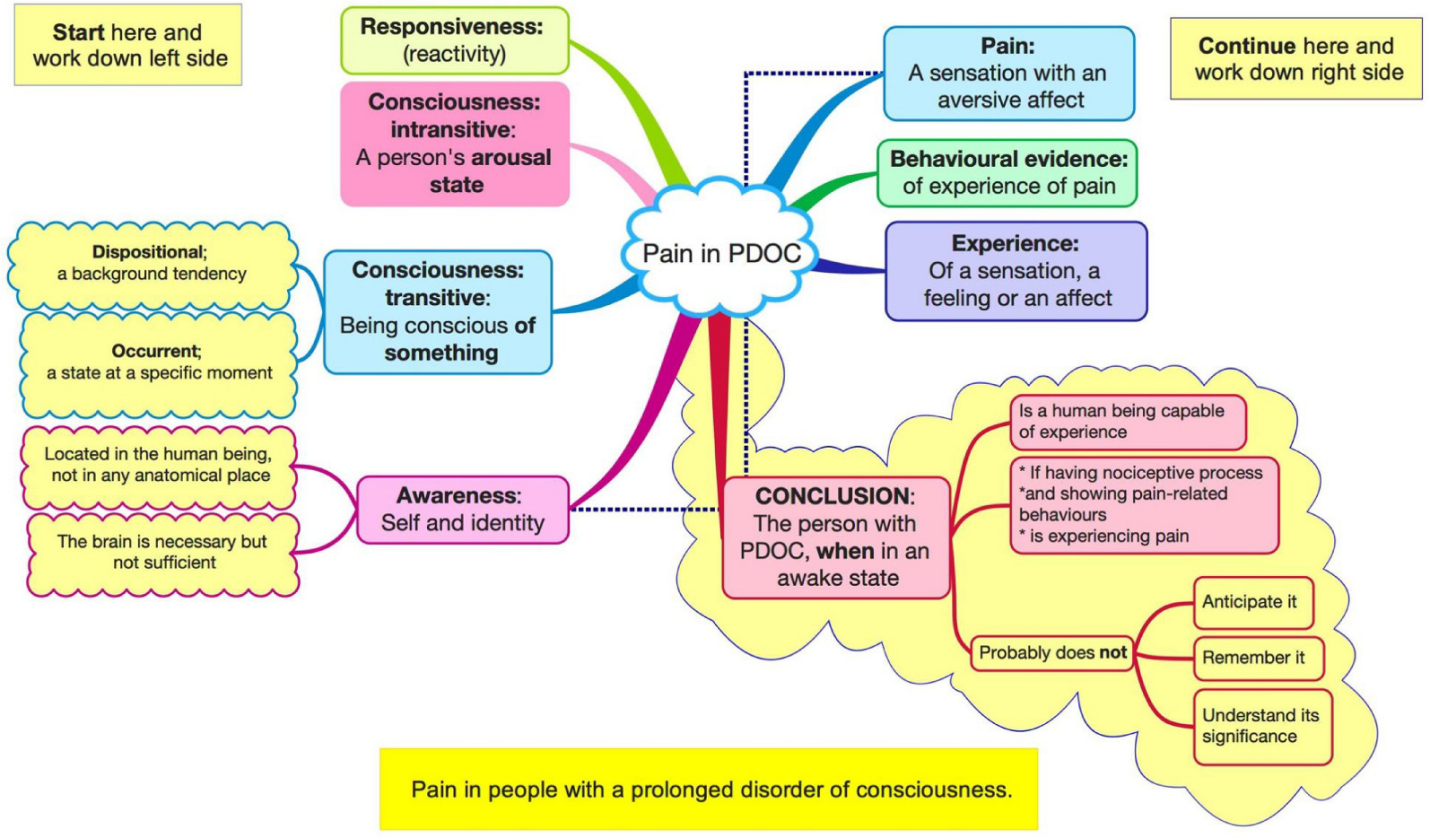
Pain in people with a prolonged disorder of consciousness (PDOC).

We searched Google Scholar and PubMed, using words such as disorders of consciousness, pain and minimally conscious state (MCS) and any papers pertinent to our investigation. This is not a systematic review. Consciousness and pain both have vast literature. Moreover, pain is a subjective phenomenon that cannot be measured in people who cannot report their experience, and any research necessarily depends on judgement. The paper works through many complex conceptual issues. The general structure can be seen in Figure 1.

## Responsiveness

To respond (‘*do something as a reaction to someone or something*’ [OED]) and to react (‘*act in response to something; respond in a particular wa*y’ [OED]) are similar in meaning. In everyday use, to respond implies being willed, deliberate or purposeful, whereas to react does not, indicating an instinctive or impulsive act. We prefer response and responsiveness to reaction and reactivity but do not intend any other distinction.

All living things react at some level to external or internal stimuli. In animals, these responses manifest as altered behaviour. An amoeba will withdraw a pseudopod from a harmful item or move towards food, just as a zebra will flee from a lion or move to a waterhole. The complexity of behaviour depends on the nervous system and the evolution of inbuilt automatic behaviours, such as courtship rituals.

The knee-jerk reflex is a monosynaptic reflex that does not require consciousness. The nociceptive-induced flexion withdrawal reflex is a simple behaviour but a polysynaptic reflex. A facial grimace with increased breathing associated with nociceptive stimuli is yet more complex.

It is more accurate to refer to these reactions to stimuli as automatic behaviours. This acknowledges that they may be complex while highlighting that they do not depend on or provide evidence of consciousness.

Boxes 1.1 and 1.2 in the UK National Clinical Guidelines^9^ illustrate automatic behaviours seen in people with a prolonged disorder of consciousness; they include

- Visual pursuit (tracking) and fixation
- Auditory or visual localisation
- A startle response to loud noise
- Groaning and facial grimacing when given a stimulus that would usually cause pain
- Extending an arm when stretching
- Scratching, yawning, purposeless spontaneous movements
- Occasional and inconsistent higher-level behaviours

Reactions to stimuli vary according to the depth of unconsciousness. This is the scientific basis of the Glasgow Coma Scale^10^ and Coma Recovery Scale – Revised.^11^ Thus, reactivity is a continuous variable, with behavioural responses becoming more complex as the consciousness level increases, without definite boundaries.

## Intransitive consciousness

An understanding of the nature of consciousness is crucial. The following brief summary draws on the analysis by Bennett and Hacker^12^ and a historical and philosophical overview by Robert van Guilick.^13^ The latter outlines several meanings of consciousness: sentience (being responsive), wakefulness or arousal, self-consciousness (awareness of self), the qualitative feeling of being and being conscious of something.

Intransitive consciousness^12^ has no object and refers to arousal or the level of consciousness. This varies in all animals, with significant changes associated with sleep or being anaesthetised and less dramatic fluctuations such as drowsiness and daydreaming. This variability is inevitable and has many sources, including medications, fatigue and the environment. The variation includes hyperarousal, for example, when afraid.

Arousal is thus a continuous variable that also lacks clear boundaries. This, coupled with natural variability, means one cannot categorise a person's state;^14^ the categories of the vegetative^15^ and minimally conscious^16^ states are invalid. The natural variation also underlies the mistaken idea that there is a 40% misdiagnosis rate.^17^ The primary direct neurophysiological influence on arousal is the ascending reticular activating system.^18,19^

## Transitive consciousness

This refers to the content of consciousness; one is conscious of something. Bennet and Hacker, in Figure 10.3,^12^ suggest five domains commonly used: perceptual consciousness of something, such as the lecturer on stage; consciousness of actions one is undertaking; reflective consciousness (e.g., considering facts); consciousness about emotions; and reflective self-consciousness of, for example, one's appearance.

This aspect of consciousness refers to the *content* rather than the level. Bennett and Hacker further distinguish two mental states. A dispositional state is one in which the person is conscious of something in the background, such as generally being low in mood or knowing one has a facial scar. An occurrent state is an active focus of attention, such as being embarrassed by a facial scar when photographed.

Transitive consciousness is primarily generated in the cerebral cortices and requires cortical memory mechanisms. Bennet and Hacker argue that ‘*intransitive consciousness and perceptual transitive consciousness … are not peculiar to human beings*’.^12^

## Awareness

Awareness of the self as a being is a higher-order, meta-cognitive phenomenon largely dependent on preserved language,^12^ and it, too, is a continuous variable with no valid categories. The distinction between awareness and consciousness, if any, is a philosophical, neuropsychological and clinical challenge.^20,21^

Awareness is an extension of consciousness to self-consciousness: ‘*A self-conscious subject is aware of themselves* **as themselves***; it is manifest to them that they themselves are the object of awareness’.*^22^ The Global Neuronal Workspace Theory^23,24^ and the Integrated Information Theory^25,26^ both suggest self-consciousness arises from a higher-order cognitive processing than consciousness alone.

Bennett and Hacker^12^ consider that awareness of the self depends on language, as language allows the development and use of abstract concepts, including the idea of self. They emphasise that one does not ‘have a self’ as an independent entity. Instead, one can form a conceptual model of selfhood through concepts encoded in language. Non-human primates and other animals cannot achieve this self-awareness because they cannot create these concepts.

## Pain – background

In 2020, the International Association of the Study for Pain published its new definition of pain: ‘*An aversive sensory and emotional experience typically caused by, or resembling that caused by, actual or potential tissue injury*’.^27^

Two of the six qualifying notes are especially pertinent:

- ‘Pain is always a subjective experience that is influenced to varying degrees by biological, psychological and social factors’.
- ‘Verbal description is only one of several behaviors to express pain; inability to communicate does not negate the possibility that a human or a non-human animal experiences pain’.

They also identified three types of pain. We will only consider *nociceptive pain* arising from peripheral nociceptors and nerves that detect actual or potential tissue damage in the body.^28,29^ We are not considering neuropathic or nociplastic pain (i.e., chronic pain).^30^ The former arises from lesions in the somatosensory nervous system. The latter exists without tissue damage and is associated with changes in the default mode, salience and somatosensory cortical networks,^31^ so it is unlikely in a person with a prolonged disorder of consciousness.

### Nociception

In animals, mechanisms evolved to detect harmful stimuli and coordinate responses to avoid or reduce harm.^32–34^ Even the most primitive neural networks, for example, in hydra, detect and direct response to nociception (harm) and can do so even after damage to neural tissue.^35,36^

Nociceptive sensation acquired a negative, unpleasant value, so the animal attended to and learned from harmful situations. This mechanism is responsible for the sensation's affective (emotional) aspect; the combination leads to pain.^32,37^ It also acquired pain-related social behaviours to attract help from others nearby. In humans and other mammals, the primary example is the facial expression of pain,^38^ and tear production may be another.^39–42^

Nociception arises from peripheral nociceptors and nerve endings that detect actual or potential tissue damage in the body.^28,29^ This sensory mechanism allows animals to sense and avoid potentially tissue-damaging stimuli, which is critical for survival.

The nociceptive impulses travel to the brain through the spino-reticular tract, which influences alertness and arousal; the spino-tectal tract, which orients the eyes and head towards noxious stimuli; and the spinothalamic tract, which contains two separable tracts:

- The anterior spinothalamic tract is the conventional ‘painfulness’ pathway. It connects to the lateral thalamic nuclei (called the ‘lateral pathway’) and encodes for pain characteristics such as location, intensity and type. It projects to the somatosensory cortex.
- The lateral spinothalamic pathway relays to medial thalamic nuclei (called the ‘medial pathway’). This pathway encodes the affective (suffering) and motivational components of pain and suffering.^42^ It projects to the anterior cingulate cortex and anterior insular cortex.

### Nociceptive pain networks

Nociceptive sensory input triggers protective responses that evolved early and are now enacted in the brainstem and midbrain, where they are more resistant to structural damage.

Nociceptive stimuli that induce pain activate multiple brain areas throughout the whole brain, from the medulla to the cortex, often called the pain (neuro) matrix,^43,44^ but activation is not unique to pain.^45^ The parts may form a distributed system rather than a serial system.^46^ Furthermore, the changes are mainly non-specific to pain.^47^ Cortical areas modulate neural activity in lower pain pathways, for example, increasing aversive behaviour in chronic pain (in rats).^48^

The subcortical structures of the brainstem and midbrain have many nuclei and pathways reacting to nociceptive signals transmitted from the spinal cord. Spino-mesencephalic tracts carry nociceptive nerve signals to the periaqueductal grey, pre-tectal area, Darkschewitsch and cuneiform nuclei and midbrain reticular formation. The spinothalamic tract carries information to the reticular formation, other mesencephalic structures and the thalamus. Other tracts from the spinal cord also interact with the brainstem and midbrain nuclei.^49^

These pathways and nuclei are involved in modulating spinal activity and receiving modulatory influences from the cortex. Some, especially the periaqueductal grey nucleus, are involved in autonomic and motor responses.^50–52^ Multiple interconnections between nuclei within the brainstem and midbrain are activated by nociceptive signals and linked with cortical and autonomic networks.^53^ Other studies have shown that complex networks in the brainstem and midbrain are involved in processing nociceptive stimuli^54^ and pathways from the cortex modulating lower centres.^55^

This exceptionally brief overview of brain pathways and nuclei involved in processing nociceptive signals inducing reactions and behaviours illustrates three crucial features:

1. Networks activated by pain encompass all parts of the brain.
2. The brainstem and midbrain have a complex series of nuclei and tracts processing nociceptive signals, including activating some responses.
3. The cortex influences lower brain networks.

## Experiencing pain

A person's behaviour is the only way to indicate their pain. The most straightforward behaviour is for them to tell someone they have pain; if someone says they have pain, we cannot disprove their report. Pain is associated with certain behaviours. A baby who screams and grimaces or someone who winces when putting weight on an injured leg is probably experiencing pain.

The most frequent behaviours associated with presumed pain in people unable to communicate due to a severe learning disability are altered or abnormal motor movements, facial activity, non-verbal vocal expression and physiological indicators.^56^ Unconscious brain-injured critical care patients show three core behaviours associated with painful procedures: facial expressions, body movements and increased muscle tone. Less consistently mentioned features include vocalisation, ‘fighting’ the ventilator, other specific changes and physiological changes.^57^

Clinical assessments have been designed for patients who cannot communicate, such as the Abbey Pain Scale,^58^ the Nociception Coma Scale^59,60^ and a behavioural tool shown in Table 2.4 of the UK national guideline.^9^ These scales record behaviours or physiological changes.

One behaviour not often mentioned in reviews but seen in unconscious people is the presence of tears. The neural pathways underlying emotional tear production are integrated into networks involved in emotion and other aspects of communicating distress, such as vocalisation and facial expression. The brain areas involved include the pons and brainstem, the periaqueductal grey matter, the cerebellum and the central autonomic network.^39–42^ Nevertheless, tears can also be seen when the clearance of lubricating tears is impaired, so they do not definitively indicate an emotional state. Thus, nociceptive stimuli in unconscious people are associated with specific behaviours, tear production and other autonomic changes.

### Experience: What and where?

The experience of pain can be considered one aspect of bodily awareness, where one must account for ‘*the sensory component of pain, which represents the felt location and intensity of the sensation, and for the affective component, its unpleasantness, which motivates protective behaviors*’.^61^ We now discuss the nature of experience and where it is located. This discussion is mainly based on Bennett and Hacker, chapter 11.^12^

Conscious experience is a mental state with two components: a perception of the sensation, which has individual characteristics, and an associated affective state. For example, stretching a spastic muscle will be felt in the muscle and might be described as deep or tearing by a conscious person. This would be specific to the muscle and situation. However, the affective response is an occurrent state with a negative hedonic tone; it is unpleasant. This will be the same for whatever spastic muscle is stretched.

Thus, the primary aspect of consciously felt pain is being in an unpleasant mental state. Its anatomical origin and specific characteristics are secondary; they enable a purposeful reaction when someone has a sufficient cognitive function, which is not available to an unconscious person.

Where is the experience located? Typically, people answer either in the muscle (‘my muscle hurts’) or the brain (‘pain is felt in the brain’). This reasoning is an example of a *mereological fallacy*: ‘… *the mereological fallacy … ascribes psychological predicates to parts of an animal that apply only to the (behaving) animal as a whole*’.^12,62,63^ As Bennett and Hacker demonstrate, the experience is felt by the being, in this case, a human being. The muscles, nerves and brain are all actively involved in producing the experience; none is its location.

The central point is that *the Person experiences pain*. The brain is necessary as a part of experience, but experience belongs to the whole body, not just a single part. For example, pain felt in a limb that has been amputated (phantom limb pain) cannot be localised in the (now absent) limb. This logic explains why brain activation studies (e.g., functional magnetic resonance imaging) cannot indicate whether an unconscious person is *experiencing* pain; they may inform probability estimates.

## Prolonged disorders of consciousness

In 2020, the Royal College of Physicians working party on managing people with a prolonged disorder of consciousness defined it as: ‘*any disorder of consciousness that has continued for at least four weeks following sudden onset brain injury’*.^7^ It includes coma and the vegetative and minimally conscious states. Most patients with prolonged disorders of consciousness have progressive neurological conditions such as multiple sclerosis, Huntington's disease or dementia.^64,65^ Cumulative co-morbidities accrued over a while may also result in a prolonged disorder of consciousness. This article refers to all those people with prolonged disordered consciousness resulting from all these conditions.

Brains from patients who had a prolonged disorder of consciousness show widespread cortical damage, diffuse axonal injury after trauma and extensive nerve cell body loss after metabolic injury, combined with damage to the dorsomedial and ventral posterior thalamic nuclei.^66,67^ Thus, pain networks in the cortex are inevitably extensively disrupted, and it is unlikely that any cognitive analysis or moderation of lower brain networks will be undertaken.

Midbrain and brainstem structures are more resistant to damage.^66^ Less extensive damage will not disrupt lower networks because networks are intrinsically resilient and resist degradation unless the loss is severe.^68^

Therefore, lower brain networks are probably still functioning in people with prolonged disorders of consciousness. The nature of the output and how the loss of cortical modulation of their function affects it are unknown, but they may increase arousal and produce non-specific non-willed behaviours. These aversive behavioural and autonomic reactions are retained as basic outputs arising from nociceptive stimuli.

Many factors are likely to lead to pain in people with a prolonged disorder of consciousness:^44,60^

- Nursing and care procedures, such as moving and handling people, stretching muscles to facilitate personal care, tracheal suctioning, complex mouth care, bowel management and replacing urethral catheters.
- Muscle immobility and increased muscle tone, maintaining posture and positioning for long periods
- Blood tests and injections for spasticity and salivary management
- Other unpredictable phenomena such as hypersensitivity to touch, muscle spasms, local infections, skin pressure ulcers, hiccups and coughing.

Furthermore, an unconscious person:

- has functional nociceptive receptors and peripheral nerves so signals reach the spinal cord and brain.
- Often shows behaviours commonly associated with pain.
- May show reduced pain behaviours after analgesia.

Nevertheless, people with a prolonged disorder of consciousness do not show more complex behaviours, such as attempting to avoid, reduce or stop the unpleasant stimulus or its cause. The observed pain behaviours are automatic and do not depend on cortical activity.

Lionel Naccache's analysis of the minimally conscious state^69^ highlighted the challenges associated with prolonged disorders of consciousness:

- ‘*cortical activity and cortically driven behaviours are not specific to conscious states*’.
- ‘*conscious states … require, instead, a brain-scale communication that has to be sustained, complex and differentiated*’.
- ‘*… MCS covers a large and heterogeneous set of states that may span from unconscious patients with residual islets of cortical activity that translates into overt behaviour, to conscious but cognitively impaired patients …*’

The definition of emergence from the minimally conscious state (MCS) has two criteria: functional use of objects and functional communication.^16^ Table 1.5 in the National Clinical Guideline^9^ considers people have a prolonged disorder of consciousness until they consistently and repeatedly:

- Use objects functionally
- Discriminate consistently between two pictures or objects
- Show evidence of self-awareness
- Show proof of meaningful awareness of their environment

Thus, people with a prolonged disorder of consciousness cover a wide range from virtually no reactions to periods of being quite reactive.

## The dilemma

Do some people with a prolonged disorder of consciousness experience an aversive state and suffer? A review of neuroimaging studies found more cortical activation associated with increasing levels of reactivity, saying, ‘*These findings suggest that the perception of pain increases with the level of consciousness and that patients with MCS can experience pain to some extent …*’.^7^ As Mr Justice Cobb observed:^70^ ‘*i.e., there are, plainly, many degrees of consciousness (from those who are only just above vegetative to those who are bordering on full consciousness) within the broad category of “MCS”’*.

Consider that patients with a prolonged disorder of consciousness:

- May still have some residual functioning cortex.^69,71^
- Have frequent procedures that may stimulate nociceptors
- Will generate nociceptive signals to activate intact subcortical centres and pathways
- Can be awake (aroused), especially when given a nociceptive stimulus
- Often show pain behaviours associated with pain-inducing processes
- May show fewer pain behaviours when given analgesic drugs.

## Synthesis

People with a prolonged disorder of consciousness typically show a sleep–wake cycle, although this is not observed in a coma. They have periods of intransitive consciousness but become aroused or awake at times and may have forms of consciousness when aroused.

Their loss of cortical function has several consequences. Their disrupted memory causes a loss of narrative identity and, with the lack of analytic processing, a coherent understanding of the environment. They will not have perceptual consciousness. The associated loss of language and the ability to form and use concepts leads to a loss of self-awareness and reflective consciousness. They inhabit a transient world lacking meaning.

On the other hand, their preserved primary sensory pathways and lower brain networks enable reactions to stimuli. They may react to loud, harsh noises, cold, flashing lights, etc., and may exhibit a ‘relaxation response’ to soothing and familiar noises, lights, etc. They may also have rudimentary mental states, albeit considerably different from before they lost consciousness.

Thus, this article proposes that they may have episodes of transitive occurrent (‘in the moment’) consciousness of mental states when awake.

This state is likely to be an unpleasant one with a negative experience of pain. They could have rudimentary but sufficiently available pain networks to generate behaviours expressing the aversive experiences and suffering typically observed in conscious people in pain. Three postulated processes are likely to be of importance: (a) The loss of cortical inhibitory influences may affect the experiential nature of nociceptive stimuli, (b) markedly reduced cortical processing will remove the meaning of this experience and (c) severe memory impairment will mean the experience is not recalled when the nociceptive input ends.

## Discussion

We acknowledge the possibility that someone with a prolonged disorder of consciousness is likely to be experiencing pain when they show typical pain behaviours. The experience is likely to be unpleasant, carry no additional meaning, and not recalled once the nociceptive stimuli end. The nature and quality of this experience will likely differ from that previously experienced when conscious. Conversely, we cannot know whether the absence of typical pain behaviours necessarily means the person is not experiencing pain.

The main reasons for our conclusion are:

- Nociceptive receptors and pain networks arose in animals very early in evolution
- Nociceptive and pain networks are:
  - Extensive throughout all levels of the brain, especially lower brain structures
  - Physically resistant to damage in the lower brain
  - Resilient in the face of damage
- Complex pain behaviours are seen in people with prolonged disorders of consciousness exposed to pain-generating stimuli.
  - These behaviours are not dependent on cortical integrity
  - They are reduced by treatments that reduce pain

The uncertainty around these matters cannot be resolved because pain is always a subjective experience. There is no alternative way to know this when a person cannot communicate.

Four crucial consequences follow when managing anyone with a prolonged disorder of consciousness:

- Standard definitions and arbitrary categories within prolonged disorders of consciousness suggest the complete absence of awareness and experience. Still, behaviours suggesting the patients have a negative experience must be actively investigated and managed, and even if pain behaviours are not seen, all nociceptive stimuli must be minimised.
- People who exhibit pain behaviours should be treated to reduce predictable pain and minimise apparent pain.
- The probability of experiencing pain should be a relevant factor when deciding the patient's best interests.
- One must anticipate and manage the distress this analysis will likely cause family, friends and the clinical team involved with the patient.

**Clinical messages**

In people with a prolonged disorder of consciousness

- pain behaviours are likely to have an unpleasant, aversive experience at the time.
- Potentially painful procedures should always be minimised
- prophylactic analgesia should be used if appropriate.
